# An anisotropic parameterization scheme for longwave irradiance and its impact on radiant load in urban outdoor settings

**DOI:** 10.1007/s00484-023-02441-3

**Published:** 2023-02-24

**Authors:** Nils Wallenberg, Björn Holmer, Fredrik Lindberg, David Rayner

**Affiliations:** Department of Earth Sciences, University of Gothenburg, 413 20 Gothenburg, Sweden

## Abstract

A robust representation of the radiative properties in complex urban settings is important for accurate estimations of radiant load. Here, we present a new parameterization scheme in the SOlar and LongWave Environmental Irradiance Geometry (SOLWEIG) model that partitions the upper hemisphere into 153 patches. Partitioning of the upper hemisphere enables determination if longwave irradiance originates from the sky, vegetation, sunlit building surfaces, or shaded building surfaces from each patch. Furthermore, a model for anisotropic sky longwave irradiance where emissivity increases with zenith angle is included. Comparisons between observations and simulations show high correlation, with *R*^2^ and RMSE for *T*_mrt_ of 0.94 and 4.6 °C, respectively, and *R*^2^ and RMSE for longwave radiation of 0.89 and 14.1 Wm^−2^, respectively. Simulations show that mean radiant temperature (*T*_mrt_) can be up to 1.5 °C higher with an anisotropic sky compared to a uniform sky as an effect of higher radiant load on the vertical of a human when sky longwave irradiance increases with zenith angle. In comparisons of simulated *T*_mrt_ with the new parameterization and old parameterization schemes, previously overestimated *T*_mrt_ under trees (high sky obstruction, sky view factor (SVF) < 0.3) can be decreased by up to 3 °C from more realistic estimations using the patches. Moreover, *T*_mrt_ close to sunlit walls (SVF ~ 0.5) is increased by up to 2–3 °C from increased exposure to sunlit surfaces. Concluding, anisotropic sky longwave radiation and directionality of longwave radiation from different sources are important in estimations of *T*_mrt_ of humans in outdoor settings.

**Supplementary Information:**

The online version contains supplementary material available at 10.1007/s00484-023-02441-3.

## Introduction

The generally warmer urban climate that results from building density, street orientation, color of materials, absence of permeable surfaces, and lack of vegetation (Arnfield 2003) puts the urban population at a greater risk of mortality and morbidity during excessive heat events (Dousset et al. 2010; Gabriel and Endlicher 2011). While the effects described by Arnfield (2003) mainly refer to nighttime, the above-mentioned factors also influence daytime microclimate, with likewise negative effects on humans (e.g., Thorsson et al. 2014). This illustrates the importance of appropriate human thermal comfort models, e.g., RayMan (Matzarakis et al. 2007), ENVI-met (Bruse and Fleer 1998), and SOlar and LongWave Environmental Irradiance Geometry model (SOLWEIG (Lindberg et al. 2008)), for urban planning in relation to urban outdoor settings.

Radiation from the sky vault is usually represented by the three components of shortwave direct and diffuse irradiance and longwave irradiance. A realistic representation of shortwave diffuse sky radiation (hereafter referred to as diffuse sky irradiance) includes the effects of circumsolar and horizon brightening, i.e., that diffuse sky irradiance originating from around the sun (circumsolar) and close to the horizon (horizon brightening) are brighter compared to other parts of the sky. These effects are omitted in isotropic sky models. The relevance of a realistic representation of diffuse sky irradiance in estimating mean radiant temperature (*T*_mrt_) for a human was demonstrated by Wallenberg et al. (2020). Their results show that the implementation of an anisotropic model for diffuse sky irradiance in SOLWEIG (Lindberg et al. 2008) led to increased radiant load on a human compared to a uniform isotropic sky.

Down-welling longwave irradiance is also often considered to be isotropic when modeling radiant load on humans. Common practice is to use models for global sky emissivity (e.g., Ångström 1915; Berdahl and Martin 1982; Prata 1996) from which sky longwave radiation is estimated. Down-welling longwave irradiance depends on the emissivity and temperature of the sky vault. That emission of longwave radiation from the sky is anisotropic has been known for almost 100 years (e.g., Dines and Dines 1927; Elasser 1942; Robinson 1947; 1950; Awanou 1998) but has hitherto, to the author’s best knowledge, not been included in modeling of the radiant load of humans. It has been demonstrated in numerous studies that emissivity increases with zenith angle, reaching its maximum close to the horizon, particularly on clear days (Bliss 1961; Unsworth and Monteith 1975; Unsworth 1975; Martin and Berdahl 1984a, b; Nahon et al. 2019). The higher emissivity from lower parts of the sky vault influences estimations of absorbed energy on vertical surfaces, which have effects on, e.g., human thermal comfort. Nahon et al. (2019) evaluated the models by Bliss (1961) and Martin and Berdahl (1984a) and found a high correlation between field observations from France with the model by Martin and Berdahl (1984a).

For a standing human, lateral longwave irradiance has a significant effect on T_mrt_ (Lindberg et al. 2013) and is mainly attributable to building surfaces that are warm compared to the sky. Nevertheless, if the lower parts of the sky vault, in the real world, are warmer, but omitted in modeling (i.e., treated as isotropic), then longwave exposure on the vertical surfaces of a human will be underestimated.

To model anisotropic diffuse sky irradiance, Robinson and Stone (2004) combined a radiation model for anisotropic diffuse sky irradiance by Perez et al. (1993) with the division of the sky vault into 145 patches, developed by Tregenza (1987), into a simplified radiosity algorithm (SRA). In a similar method, Robinson and Stone (2005) implemented a model for isotropic sky longwave irradiance estimated from dew point temperature, partitioned into the 145 patches described above. Rykaczewski et al. (2021) referred to the differentiation of global shortwave radiation into direct and diffuse components as anisotropy, following the methods by Holmer et al. (2015). The anisotropy in this method is the direct component, which is possible to estimate together with the position of the sun (zenith and azimuth angles), whereas the diffuse component is still considered isotropic. The separation into direct and diffuse components improved model results. The authors explained this by the fact that some parts of the manikin were obstructed from the direct solar beam and only exposed to diffuse sky irradiance as opposed to simulations using global shortwave radiation. Nevertheless, since their simulated radiant load was for an unobstructed setting (rooftop) with uniform conditions for diffuse sky irradiance and longwave radiation, they concluded that further model evaluation should be performed in complex urban settings using anisotropic diffuse sky irradiance and anisotropic longwave radiation.

SOLWEIG (Lindberg et al. 2008, 2016; Lindberg and Grimmond 2011; Wallenberg et al. 2020) is a frequently used model for radiant load on humans (e.g., Lindberg et al. 2013; Thom et al. 2016; Bäcklin et al. 2021), accessed through the Universal Multi-scale Environmental Predictor (UMEP (Lindberg et al. 2018)). In SOLWEG, T_mrt_ is estimated from 2.5D pixel-based input data for buildings (Digital Surface Model (DSM)) and meteorological data (global shortwave radiation, air temperature, and relative humidity). Optionally, a canopy digital surface model (CDSM) with vegetation height and information on ground cover can be included. SOLWEIG has been evaluated in several studies (Lindberg et al. 2008; Lindberg and Grimmond 2011; Lindberg et al. 2016; Lau et al. 2015; Chen et al. 2016; Kantor et al. 2018; Gal and Kantor 2020). In its current version, sky longwave irradiance is considered isotropic and is estimated from sky view factors (SVF) and global emissivity (Prata 1996). However, as with the SRA (Robinson and Stone 2004; 2005) for diffuse sky irradiance, partitioning of the sky vault should enable a more realistic realization of the sky longwave radiation and hence improve estimations of radiant load on humans.

In this paper, we adapt the idea by Robinson and Stone (2004; 2005), dividing the sky vault into a number of patches, according to Tregenza (1987). We estimate the effects of the Martin and Berdahl (1984a) anisotropic model for sky vault emissivity on T_mrt_. Additionally, vegetation and sunlit and shaded building surfaces are included in the patches.

## Methods

### Previous estimations of longwave radiation fluxes in SOLWEIG

In the previous version of SOLWEIG, down-welling and lateral longwave irradiance is considered isotropic and is estimated from SVF on a human represented by a standing box (see Lindberg et al. (2008; 2016) for details). Furthermore, sunlit building surfaces were estimated from a fraction of sunlit surfaces (see Fig. 3 in Lindberg et al. (2008)), which resulted in discrepancies close to sunlit building surfaces (e.g., Gal and Kantor (2020)).

### Implementation of a longwave anisotropic sky in SOLWEIG

The methods presented here follow the approach by Robinson and Stone (2005), but with an anisotropic sky according to Martin and Berdahl (1984a), where emissivity increases with zenith angle. However, we have increased the number of patches from 145 to 153 to have patches that are more similar in size, as longwave irradiance from each patch depends on patch solid angle. A too large difference in solid angle between patches could otherwise have a larger effect than the difference in emissivity. The patches are arranged in eight annuli, and each annulus is divided into n_i_ patches. The solid angle (steradian, sr) of an annulus (${A}_{\mathrm{annulus}}$) is calculated from the solid angle of a dome as the difference between domes limited by the upper (${\alpha }_{\mathrm{upper}}$) and lower (${\alpha }_{\mathrm{lower}}$) zenith angles of the annulus:

1 $${A}_{\mathrm{annulus}}=2\pi \left(\mathrm{cos}{\alpha }_{\mathrm{upper}}-\mathrm{cos}{\alpha }_{\mathrm{lower}}\right)$$

The dome solid angle of the centroid zenith angle of an annulus is given by adding half the solid angle of the annulus to the dome solid angle of the upper zenith angle:

2 $${A}_{c}=1-\left({A}_{\mathrm{upper}}+{A}_{\mathrm{annulus}}/2\right)/\left(2\pi \right)$$

Then, the centroid zenith angle (*α*_*c*_) is

3 $$\alpha_c=\arccos\;A_c$$

The contribution from an annulus to a horizontal surface at ground level (*P*_*c*_) is

4 $$P_c=A_c\bullet\cos\;\alpha_c$$

where the sum of *P*_*c*_’s from all annuli will become a solid angle of *π*.

For a standing human, the contribution to the vertical surfaces on the human body will become

5 $$Q_c=A_c\bullet\sin\;\alpha_c$$

**Fig. 1:**
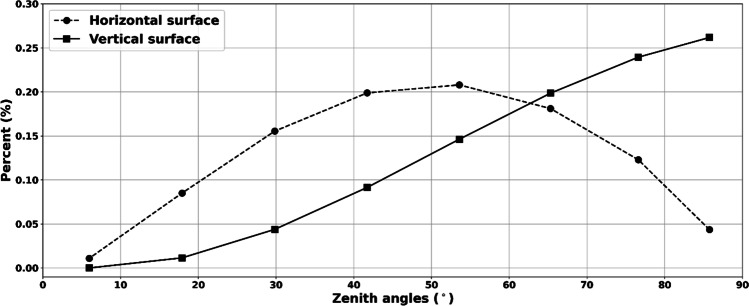
Percentage shares of down-welling fluxes from the sky on horizontal and cylindrical vertical surfaces

Details on the properties of the patches are given in Table 1, and Fig. 1 gives the distribution of weights for horizontal and vertical cylindrical surfaces.

**Table 1:** Detailed information on patch distribution and properties, where zenith angle interval is the height of each annulus/patch and centroid zenith angle is the corresponding center of each annulus/patch. Annulus solid angle is the steradian (sr) of each annulus, which divided by the number of patches gives the solid angle ($\varphi$) of each patch

Zenith angle interval (°)	Centroid zenith angle (°)	Annulus solid angle (sr)	Number of patches	$\varphi$(sr)
0–6	4.2	0.034	1	0.034
6–18	13.4	0.273	7	0.039
18–30	24.3	0.534	13	0.041
30–42	36.6	0.772	19	0.041
42–54	48.3	0.976	24	0.041
54–66	60.2	1.137	28	0.041
66–78	72.1	1.249	30	0.042
78–90	84.1	1.306	31	0.042

A vertical surface has its highest share at large zenith angles, i.e., from angles close to the horizon (Fig. 1). For a horizontal surface, the contribution from the annulus around zenith is small because of the small annulus size. Maximum is instead found at zenith angles around 40–50° since the annuli in that interval have a considerable size and the angle of incidence is still high. Closer to the horizon, the contribution again becomes small due to the low angle of incidence, even with the large annuli area.

In SOLWEIG, the expression by Prata (1996) is used to estimate the global clear sky emissivity (*ε*_sky_):

6 $${\varepsilon }_{\mathrm{sky}}=1-\left(1+46.5\frac{{e}_{a}}{{T}_{a}}\right)\bullet \mathrm{exp}\left(-{\left(1.2+3.0\bullet 46.5\frac{{e}_{a}}{{T}_{a}}\right)}^{0.5}\right)$$

where ${T}_{a}$ is the air temperature at standard height (2 m agl) and ${e}_{a}$ the actual vapor pressure in hPa calculated from standard height observations of relative humidity, where ${e}_{a}$ in SOLWEIG is estimated from ${T}_{a}$ and relative humidity ($\mathrm{RH})$.

The model by Martin and Berdhal (1984a) is used to estimate the angular emissivity (${\varepsilon }_{\theta }$), and is given by

7 $${\varepsilon }_{\theta }=1-\left(1-{\varepsilon }_{\mathrm{sky}}\right){\mathrm{exp}}^{b\left(1.7-\frac{1}{\mathrm{cos}\theta }\right)}$$

where *b* is a variable influencing the magnitude of anisotropy and θ is the zenith angle. Nahon et al. (2019) proposed to set *b* to 0.308. However, they used the model by Ångström (1915) for the estimation of global sky emissivity, whereas the model by Prata (1996) is used in SOLWEIG. Since observations of emissivity for the various parts of the sky vault are not available for this study, the constant *b* has been set to 0.308.

Thus, combining patches and anisotropy, the down-welling radiation is the sum over vertical fluxes over all patches:

8 $${L}_{\downarrow }=\sum_{i=1}^{153}{\varphi }_{i}{\xi }_{i}{{\varepsilon }_{\theta i}\sigma T}_{a}^{4}\frac{1}{\pi }$$

where $\sigma$ is the Stefan-Boltzmann constant ($5.67\bullet {10}^{-8} W{m}^{-2} {K}^{-4}$), $\varphi$ is the solid angle of the patch, and $\xi$ is the angle of incidence, where $\xi$ is calculated as follows:

9 $$\xi =\mathrm{cos}{\overline{\eta }}_{\mathrm{patch}.\mathrm{centroid}}\mathrm{cos}{\overline{\alpha }}_{\mathrm{patch}}^{^{\prime}}\mathrm{sin}\beta +\mathrm{sin}{\overline{\eta }}_{\mathrm{patch}.\mathrm{centroid}}\mathrm{cos}\beta$$

with ${\overline{\eta }}_{\mathrm{patch}.\mathrm{centroid}}$ being the altitude of the patch centroid, ${\overline{\alpha }}_{\mathrm{patch}}^{^{\prime}}$ is the azimuth angle of the patch centroid compared to the normal of a surface, and $\beta$ is the plane tilt, where 0 is a horizontal plane and $\pi /2$ is a vertical surface. Continuing on Eq. 8, $\varphi$ is estimated for the individual patches:

10 $${\varphi }_{\mathrm{patch}}=\Delta {\alpha }_{\mathrm{patch}}(\mathrm{sin}{\eta }_{\mathrm{patch}.\mathrm{max}}-\mathrm{sin}{\eta }_{\mathrm{patch}.\mathrm{min}})$$

where $\Delta {\alpha }_{\mathrm{patch}}$ is the azimuthal width of the patch and $\mathrm{sin}{\eta }_{\mathrm{patch}.\mathrm{max}}$ and $\mathrm{sin}{\eta }_{\mathrm{patch}.\mathrm{min}})$ represent the maximum and minimum altitude of the patch.

If there are clear skies and $SVF= 1$, then ${L}_{\downarrow }= {{\varepsilon }_{\mathrm{Prata}}\sigma T}_{a}^{4}$.

#### Influence of cloudiness

During cloudy conditions, Unsworth and Monteith (1975) found that the emissivity increased but that the relative impact of the zenith angle did not change. They explained this as an effect of that most of the radiation reaching the ground emanates from a 100 m air layer where CO_2_ and water vapor creates the impact of the zenith angle. On the other hand, higher up the droplets at the cloud base increase the emissivity to unity.

To estimate the influence of clouds on hemispheric emissivity, Unsworth and Monteith (1975) used

11 $${\varepsilon }_{\mathrm{sky}.\mathrm{clouds}}=\left(1-0.84c\right)\bullet {\varepsilon }_{\mathrm{sky}}+0.84c\bullet {\varepsilon }_{\mathrm{clouds}}$$

where *c* is the share of the sky covered by clouds and ${\varepsilon }_{\mathrm{clouds}}$ the emissivity of the clouds (= 1).

If cloud observations are not available, cloudiness can be estimated by a clearness index (CI) (Crawford & Duchon 1999), calculated as the ratio of observed ground-level solar radiation to clear sky ground-level solar radiation.

Crawford and Duchon (1999) calculated a fractional cloud cover (*c*) as

12 $$c=1-CI$$

and thus, the influence of clouds on the hemispherical emissivity will be

13 $${\varepsilon }_{\mathrm{sky}.\mathrm{clouds}}=\left(1-c\right)\bullet {\varepsilon }_{\mathrm{sky}}+c\times {\varepsilon }_{cl}=CI\bullet {\varepsilon }_{\mathrm{sky}}+(1-CI)\bullet {\varepsilon }_{\mathrm{clouds}}$$

When looking at CI on fully overcast days, it shows that CI will not be zero but instead about 0.2–0.3. As a result, *c* will be 0.7–0.8 on overcast days instead of 1 if based only of the cloud fraction. However, if compared with the expression by Unsworth and Monteith (1975), the factor 0.84c with $c=1$, i.e., overcast correspond to $1-CI=0.7-0.8$ obtained for overcast days. Thus, the reduced effect of cloud cover on hemispherical emissivity observed by Unsworth and Monteith (1975) fits well with that of CI for overcast equal to 0.2–0.3.

#### Building, vegetation, and reflected longwave radiation components

The shadow casting methodology in SOLWEIG (Ratti and Richens 2004; Lindberg and Grimmond 2010, 2011) can determine whether a pixel within a model domain is shaded by vegetation or a building. Utilizing the shadow casting algorithm and the centroid of a patch, it is possible to establish if a patch represents unobstructed sky, vegetation, or a building wall. In previous versions of SOLWEIG, the fraction of sunlit walls was estimated using a fictitious basin based on SVF (Fig. 3 in Lindberg et al. 2008). However, since it was estimated from the SVF of a pixel, no information about in which direction the buildings were located was included. This led to an inconsistency in that all directions had sunlit surfaces regardless of the position of the sun. A new scheme for shaded and sunlit building walls is presented in this paper, where SVF is recalculated to an average building height for a fictitious basin. With this average building height, the position of the sun, and the position of a patch determined as a building wall, it is possible to approximate if it is sunlit or not. This is determined by estimating how far down the wall in the fictitious cylindrical yard the sun reaches and comparing this with the position of the patch. The difference in surface temperature between sunlit and shaded vegetation is minor with negligible effect on T_mrt_ (Lindberg and Grimmond 2011) and is therefore treated the same regardless if the vegetation is sunlit or not.

Longwave radiation from building surfaces and vegetation (treated as shaded wall) as well as a reflected component depending on ${L}_{\downarrow }$ (Eq. 8) and ${L}_{\uparrow }$ (see Lindberg et al. (2016)) components are estimated according to previous calculations in SOLWEIG, but modified to include $\xi$ (Eq. 9) and $\varphi$ (Eq. 10) instead of weights, here exemplified for one patch:

14a $${L}_{\mathrm{WALLshadow}}={\varepsilon }_{w}\sigma {T}_{\mathrm{wall}.\mathrm{sh}}^{4}\bullet \varphi \bullet \xi \bullet \frac{1}{\pi }$$

14b $${L}_{\mathrm{WALLsun}}={\varepsilon }_{w}\sigma {T}_{\mathrm{wall}.\mathrm{sun}}^{4}\mathrm{cos}{\eta }_{\mathrm{sun}}\bullet \varphi \bullet \xi \bullet \frac{1}{\pi }$$

14c $${L}_{\mathrm{REFLECTED}}=\left({L}_{\downarrow }+{L}_{\uparrow }\right)\left(1-{\varepsilon }_{\mathrm{wall}}\right)\bullet \varphi \bullet \xi \bullet \frac{1}{\pi }$$

The new parameterization scheme, thus, consists of Eq. 8 (sky), Eq. 14a (shaded building surface or vegetation), and Eq. 14b (sunlit building surface). The reflected longwave radiation component, according to Eq. 14c, is added if the patch is a building surface or vegetation. $\xi$ determines if the radiation is received by a horizontal or a vertical surface. From this, longwave radiation originating from the upper hemisphere can be described with the following expression:

15 $${L}_{\downarrow }=\sum_{i=1}^{153}{{\varepsilon }_{p.i}\sigma T}_{p.i}^{4}{\varphi }_{i}\mathrm{cos}{\xi }_{i}\frac{1}{\pi }$$

where ${\varepsilon }_{p.i}$ is the emissivity of the patch (sky or wall), ${T}_{p.i}^{4}$ is the temperature of the patch (sky (T_a_), vegetation (T_wall.sh_), shaded building (T_wall.sh_), or sunlit building (T_wall.sun_)), as seen from a pixel in SOLWEIG.

**Fig. 2:**
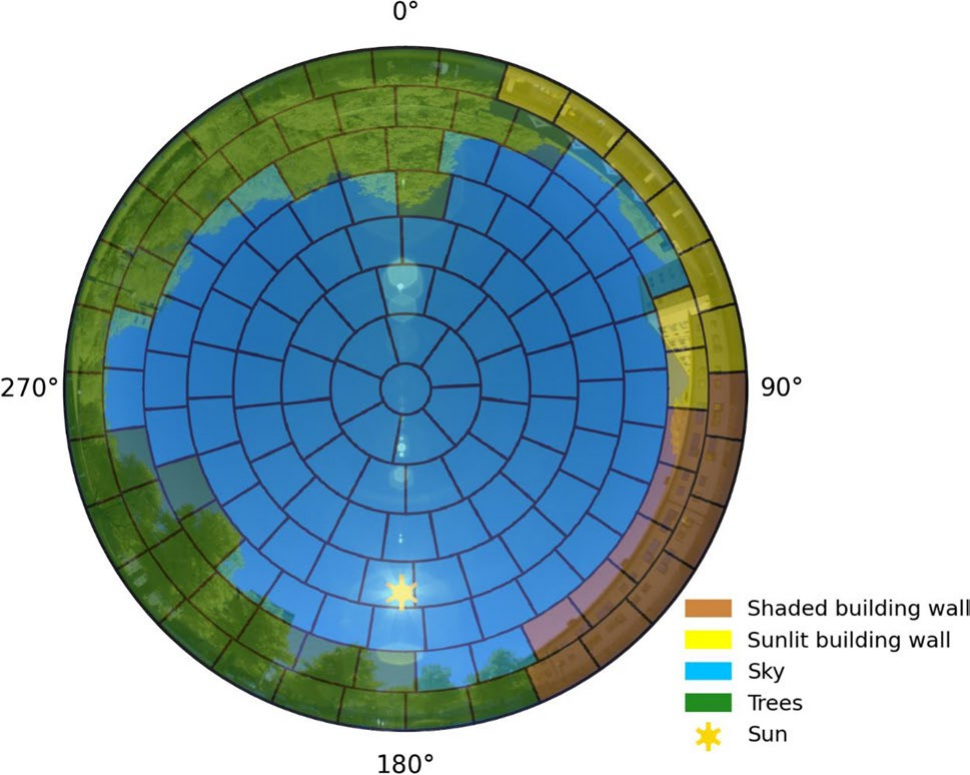
Sky view image overlaid with patches showing patch characteristics in SOLWEIG. Example is from 2021–09-02 13:20 LST, at the location shown in Fig. 3 (white star). The sky view image was captured with a Sigma 4.5 mm *f*/*2*.8 circular fisheye lens with equisolid projection. The model produced patch characteristics uses an equidistant projection, reprojected to an equisolid projection using the methods by Honjo et al. (2019) for this illustration

**Fig. 3:**
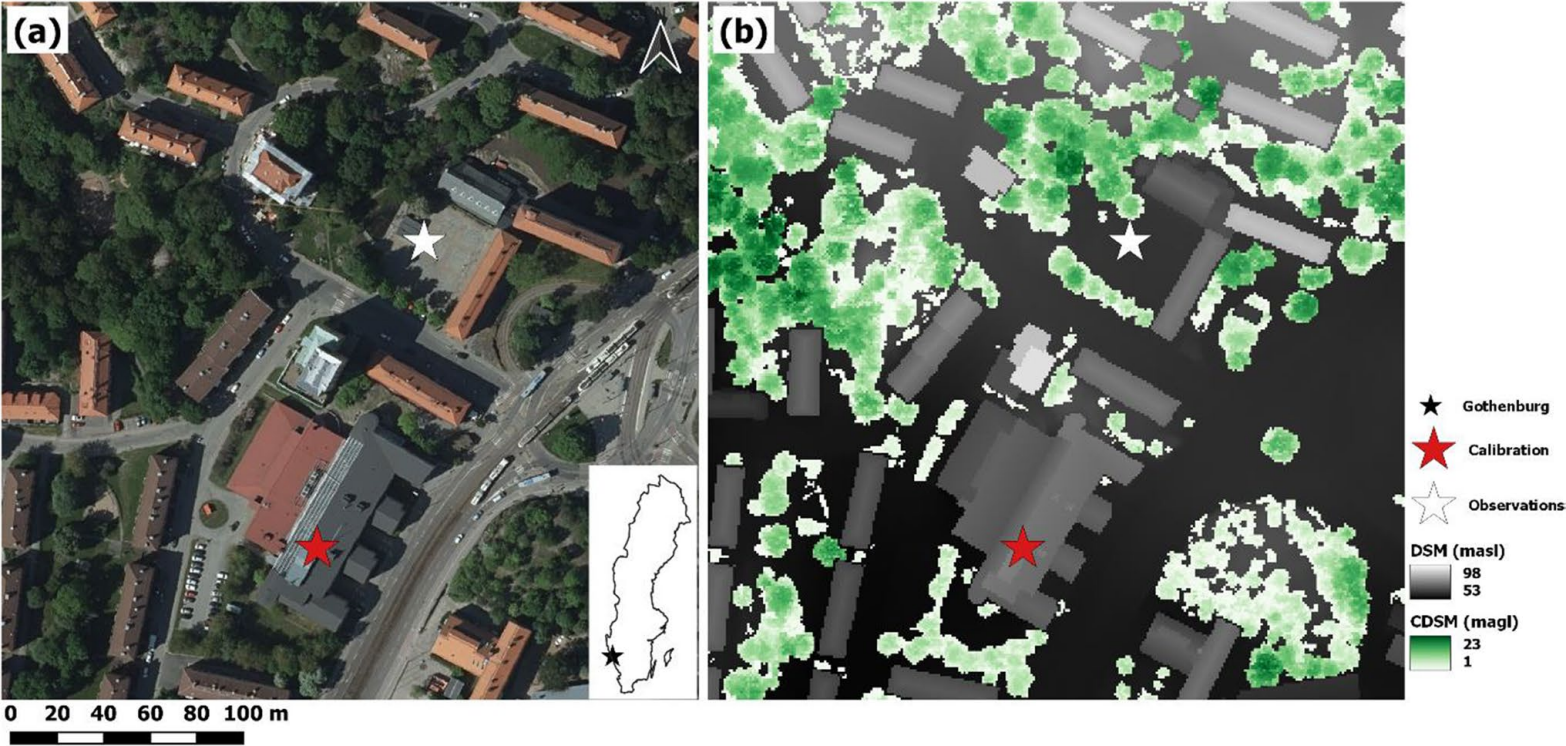
Satellite image (© Lantmäteriet) of the study area and inset with the location of Gothenburg in Sweden (made with Natural Earth) (**a**) and (**b**) corresponding digital surface model (DSM) and canopy digital surface model (CDSM). The white star indicates the location of field observations, and the red star is the weather station where the pyrgeometers and pyranometers were calibrated

A sky view image overlaid by an output from SOLWEIG showing patches categorized as sky, vegetation, shaded building wall, or sunlit building wall is presented in Fig. 2. The sky view image was captured with a Nikon D5100 camera equipped with a Sigma 4.5 mm *f*/2.8 circular fisheye lens utilizing an equisolid projection. With an equisolid projection, every pixel has an equal solid angle, compared to an equidistant projection where angular (zenith) distances are equal. The image with patch categories is reprojected from an equidistant to an equisolid projection according to Honjo et al. (2019). The location of the sky view image and SOLWEIG patch categories output are from the same location (observations (white star) in Fig. 3). The image is from 2021–09-02 13:20 LST and shows that the patch categories established in SOLWEIG give a good approximation of whether a patch is sky, vegetation, shaded building wall, or sunlit building wall. For example, the sunlit building wall patches have a difference in azimuth angle > 90° compared to the azimuth angle of the sun. Vegetation, likewise, shows a good match, with some slight offset for some pixels. The CDSM is from October 2010, which possibly explains these offsets (tree growth and/or removed vegetation and no leaves). Some building wall patches also show an offset. This can similarly be explained by discrepancies in the data, as the DSM is produced from 3D vector polygon roof structures in conjunction with a DEM, but could also be an effect of that patch characteristics are determined from the centroid of the patch.

### SOLWEIG simulation setup

Meteorological data (incoming shortwave radiation, air temperature, and relative humidity) and information on surface elevation are necessary to run SOLWEIG. The meteorological data (direct and diffuse shortwave radiation, air temperature, and relative humidity, 10 min time-step) used here was acquired from a weather station on the rooftop of the Department for Earth Sciences, University of Gothenburg (calibration (red star) in Fig. 3). Due to an instrument malfunction on the department rooftop weather station, additional meteorological data (global shortwave radiation, air temperature, and relative humidity, 60 min time-step) were acquired from a compiled meteorological dataset for Gothenburg (Rayner et al. 2021). Raster data with information on building and ground elevation (DSM) and vegetation height (CDSM) are from the City of Gothenburg (Fig. 3b). Furthermore, raster data on ground cover was included. Ground cover information enables differentiation of emissivity, albedo, and surface temperature parameterization between different surfaces (e.g., cobble stone, asphalt, soil, grass). Emissivity of the ground surface at the measurement site has been set to 0.95 with an albedo of 0.16. Emissivity of walls is set to 0.9. All raster data have a 1 m pixel resolution. In SOLWEIG, T_mrt_ can be calculated for a human represented by either a standing box or a cylinder. In this paper, T_mrt_ has been estimated for a human represented by a cylinder. Furthermore, an anisotropic sky for diffuse shortwave radiation (Wallenberg et al. 2020) is utilized. The only difference in the simulations is the representation of longwave radiation, wherein the new parameterization either an anisotropic or isotropic sky has been used for the patches (stated in the text). In the old version of SOLWEIG, obviously, the old parameterization based on SVF has been used. Thus, shortwave radiation is treated equally in all simulations to rule out any possible influence. Shortwave radiation fluxes for both days are available Online Resource (Online Resource 1 for 2021–06-17 and Online Resource 2 for 2021–06-08).

### Field measurements

Field measurements for detailed evaluation of the SOLWEIG model and its new longwave parameterization scheme took place on 2021–06-08 and 2021–06-17 at Guldhedstorget in Gothenburg, Sweden (Fig. 3). 2021–06-08 was overcast for most of the day with the sun breaking through between approximately 12:00 and 14:00 and 16:00 until sunset (see Online Resource 2). The second day, 2021–06-17, was relatively clear, with partly cloudy conditions around 09:00, 11:00–11:30, 12:30–14:30, and 17:00–17:30 (see Online Resource 1). The measurements were conducted using three Kipp and Zonen CNR1 Net Radiometers (Kipp and Zonen 2009), with two pyranometers (CM3) and two pyrgeometers (CG3) each, all connected to a Campbell Scientific CR5000 logger. Furthermore, one Delta-T SPN1 Sunshine Pyranometer (Wood 2019) was used and connected to a Campbell Scientific CR1000 logger. Down-welling shortwave radiation measured by the Delta-T SPN1 was used on 2021–06-08 as a consequence of instrument malfunction of the Kipp and Zonen CNR1 Net Radiometer measuring down-welling shortwave radiation. Data was sampled every 5 s, and 10 min average values were calculated for comparison with SOLWEIG simulations. Four supplementary days of field measurements from Guldhedstorget for correlations with SOLWEIG simulations were used (2018–05-15 (clear day), 2018–06-08 (clear), 2019–07-11 (clear), and 2020–06-24 (clear)). Here, 60 min average values were derived for comparison with SOLWEIG.

The Kipp and Zonen CNR1 Net Radiometers were setup according to the 3D integral radiation measurements suggested by Höppe (1992), i.e., facing down, up, north, south, east, and west. This setup captures down-welling (K_down_), outgoing (K_up_) shortwave radiation, and shortwave radiation from the four cardinal directions (K_north_, K_south_, K_east_, and K_west_), as well as emitted longwave radiation from above (L_down_), below (L_up_) and north (L_north_), south (L_south_), east (L_east_), and west (L_west_). This configuration has been used as a standard for comfort radiation measurements (e.g., Thorsson et al. 2007; Kantor et al. 2014a, b). Höppe (1992) approximated the person receiving these fluxes with a standing box with the following form factors: horizontal surfaces, i.e., top and bottom = 0.06 each, and vertical surfaces, i.e., north, south, east, and west = 0.22 for every side. However, as shown by Thorsson et al. (2007), Kantor et al. (2014a), Kantor et al. (2014b), and Holmer et al. (2015), the original calculation of T_mrt_ by Höppe (1992) results in a local minimum at noon and an overestimation some hours later. To avoid this, Holmer et al. (2015) introduced a division of monitored global radiation into direct and diffuse shortwave radiation. Then, the mean radiant flux (${S}_{str}$) can be estimated for a human represented by a cylinder as

16 $$\begin{array}{c}{S}_{str}={\alpha }_{k}\left[0.28{K}_{\mathrm{dir}.\mathrm{side}}+0.06\left({K}_{\mathrm{up}}+{K}_{\mathrm{down}}\right)+0.88{K}_{\mathrm{diff}.\mathrm{side}}\right]\\ +{\varepsilon }_{p}\left[0.88{L}_{\mathrm{side}.\mathrm{average}}+0.06\left({L}_{\mathrm{up}}+{L}_{\mathrm{down}}\right)\right]\end{array}$$

where ${\alpha }_{k}$ is the absorption coefficient for shortwave radiation (standard value 0.7) and ${\varepsilon }_{p}$ is the average emissivity of the human body (equal to the absorption coefficient for longwave radiation according to Kirchoff’s Law with a standard value of 0.97, here set to 0.95). *K*_dir,side_ is the horizontal component of the direct radiation normal to the bole area of the cylinder and *K*_diff,side_ the corresponding diffuse radiation. *L*_side, average_ is the average of the four cardinal points longwave sensors. Coefficients 0.28, 0.06, and 0.88 are the form factors of the cylinder: vertical cross-sectional area, top/bottom, and bole area.

With an estimated ${S}_{str}$, it is possible to calculate *T*_mrt_ with the Stefan-Boltzmann Law:

17 $${T}_{mrt}=\sqrt[4]{\left(\frac{{S}_{str}}{{\varepsilon }_{p}\sigma }\right)}-273.15$$

The method by Holmer et al. (2015), i.e., cylinder, has been used for all estimations of *T*_mrt_ from observed values.

The Kipp and Zonen CM3 Pyranometers (Kipp and Zonen 2009) were calibrated with a Delta-T SPN1 Sunshine Pyranometer (Wood 2019). Likewise, the Kipp and Zonen CG3 Pyrgeometers (Kipp and Zonen 2009) were calibrated with a Kipp and Zonen CGR4 Pyrgeometer (Kipp and Zonen 2018) in an unobstructed setting on the rooftop of the Department for Earth Sciences at the University of Gothenburg (calibration (red star) in Fig. 2). Calibration was conducted between 2021–09-01 through 2021–09-05.

## Results

### Model performance—comparison of model simulation versus observations

**Fig. 4:**
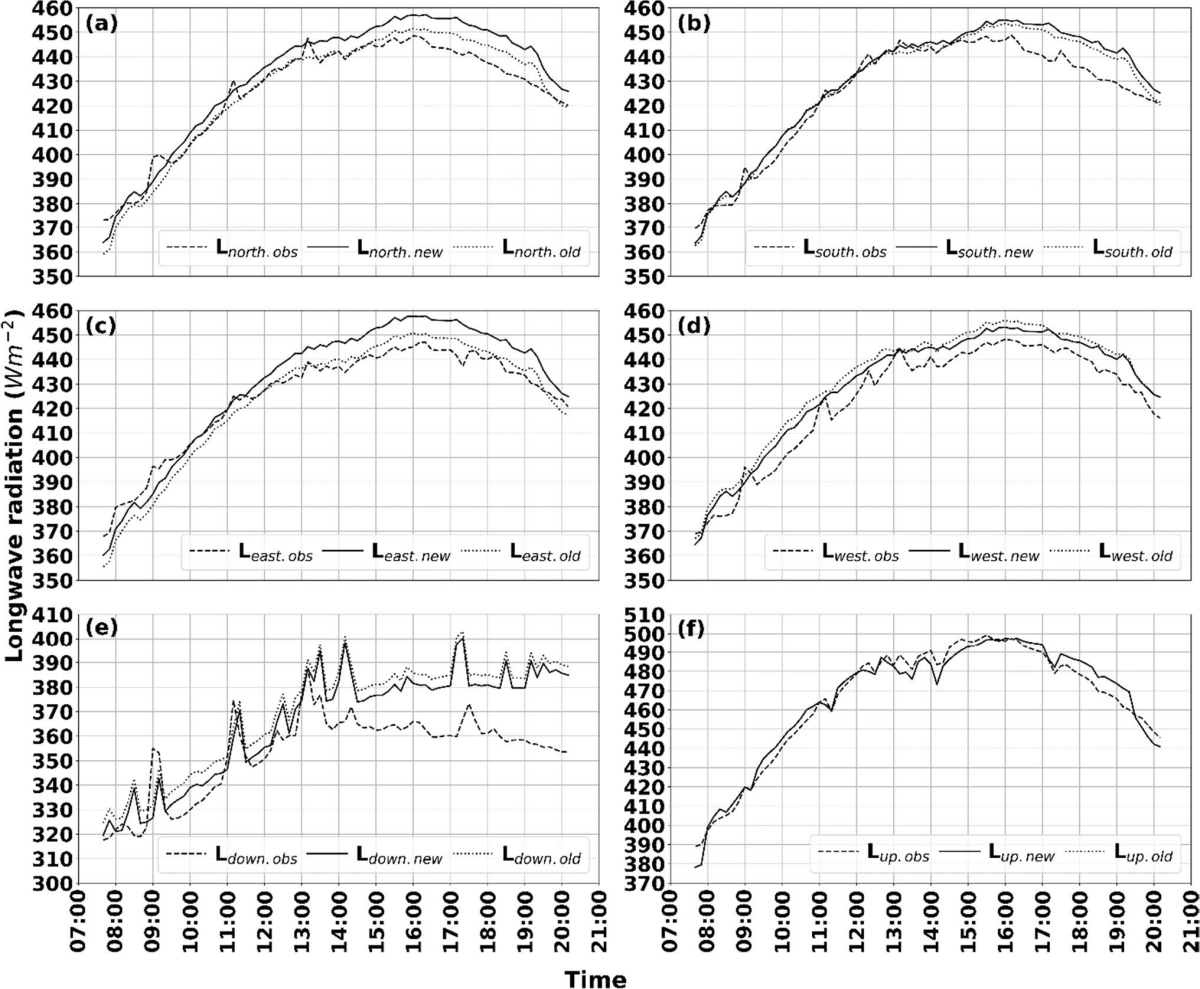
Six directional observed and simulated longwave radiation data (10 min resolution) for a relatively clear day (2021–06-17). The figure shows longwave radiation fluxes from **a** north, **b** south, **c** east, **d** west, **e** upper hemisphere, and **f** ground. Dashed lines depict observed values. Solid and dotted lines represent the new and old parameterization schemes, respectively

A comparison of observed and simulated longwave radiation with the new (anisotropic from patches) and the old (isotropic based on SVF) parameterization schemes for a relatively clear and warm day is presented in Fig. 4. Observations were carried out in a square, surrounded by both buildings and vegetation (see Figs. 2 and 3).

The figures show that differences between observed and simulated longwave radiation fluxes are relatively small throughout the day. Nevertheless, some deviations are noticeable, especially in the afternoon (Fig. 4a–e). These deviations are, however, relatively small (10–15 Wm^−2^ for cardinal directions and 10–20 Wm^−2^ for down-welling). The explanation for the deviations in Fig. 4a–d can be found in the surface temperature parameterization scheme in SOLWEIG and will be elaborated on in the discussion (see “Discussion”). Continuing, some small differences between the new and old parameterization schemes are evident mainly in the longwave radiation originating from north, south, and east. A possible explanation for this is that sunlit walls in the old scheme are always on the very top of buildings (see Fig. 3 in Lindberg et al. 2008). In the new parameterization scheme, on the other hand, walls can be sunlit from top to ground level. Thus, sunlit surfaces close to the ground will emit longwave radiation more or less perpendicular to the vertical facet of a standing human, resulting in higher radiation compared to if sunlit surfaces were at the top of a building. This explanation is supported by fluxes from the west (Fig. 4d), originating from vegetation and sky (see Fig. 2). Here, simulated fluxes are similar as there are no buildings, with the new scheme showing marginally lower values compared to the old scheme. Compared to the observed values, both simulated fluxes are overestimated, which could be because of a slightly overestimated surface temperature of the vegetation (set to *T*_*a*_). Another possible explanation for the overestimations in Fig. 4a–c is that there are some offsets in what is defined as buildings by the model (see Fig. 2). If this offset is true, it means that some of the patches that are now defined as buildings should be defined as sky—with lower emissivity (${\varepsilon }_{\mathrm{sky}}\approx 0.85$ for second annulus as compared to ${\varepsilon }_{\mathrm{wall}}=0.9$) and temperature (if wall is sunlit) values. Patch definition for this site could therefore potentially be improved with a more detailed or updated DSM.

An additional comparison was made, although not included here, between a SOLWEIG simulation with an anisotropic sky for longwave radiation and a simulation with an isotropic sky (both using patches). This comparison showed that differences for the actual location of measurements are very small, indicating that overestimations are not a product of sky longwave radiation.

**Fig. 5:**
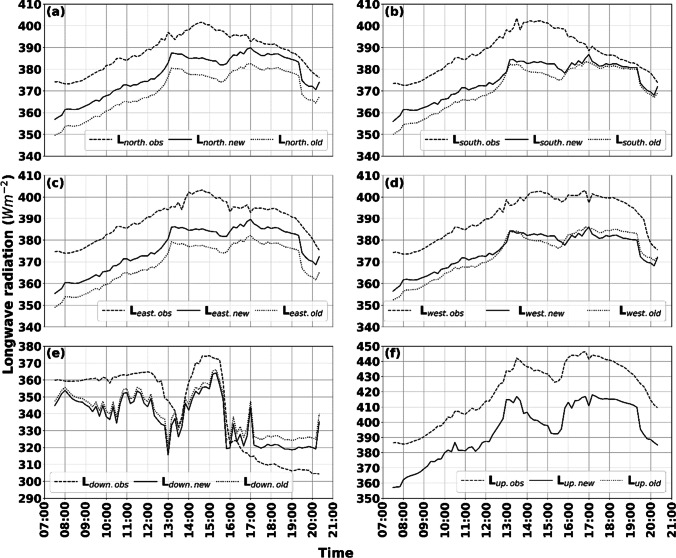
Six directional observed and simulated longwave radiation data (10 min resolution) for a relatively clear day (2021–06-08). The figure shows longwave radiation fluxes from **a** north, **b** south, **c** east, **d** west, **e** upper hemisphere, and **f** ground. Dashed lines depict observed values. Solid and dotted lines represent the new and old parameterization schemes, respectively

Results from an overcast–semi-cloudy–clear day (2021–06-08) are presented in Fig. 5. Here, it is evident that simulated longwave radiation is underestimated for both schemes. On the other hand, simulated values with the new scheme show improvements compared to the old scheme. Some of the underestimations seen in simulated fluxes can, again, be traced to the surface temperature parameterization scheme (see “Discussion”). Furthermore, ${\varepsilon }_{\mathrm{sky}}$ seems to be underestimated as simulated *L*_down_ at the weather station (calibration, red star in Fig. 3), where $SVF\approx 1$ does not equal observed L_down_ (underestimated with about 13 Wm^−2^ at 12:10, not shown). Largest difference is visible in L_up_ (approximately 30 Wm^−2^), whereas the cardinal fluxes have deviations of about 20 Wm^−2^.

**Fig. 6:**
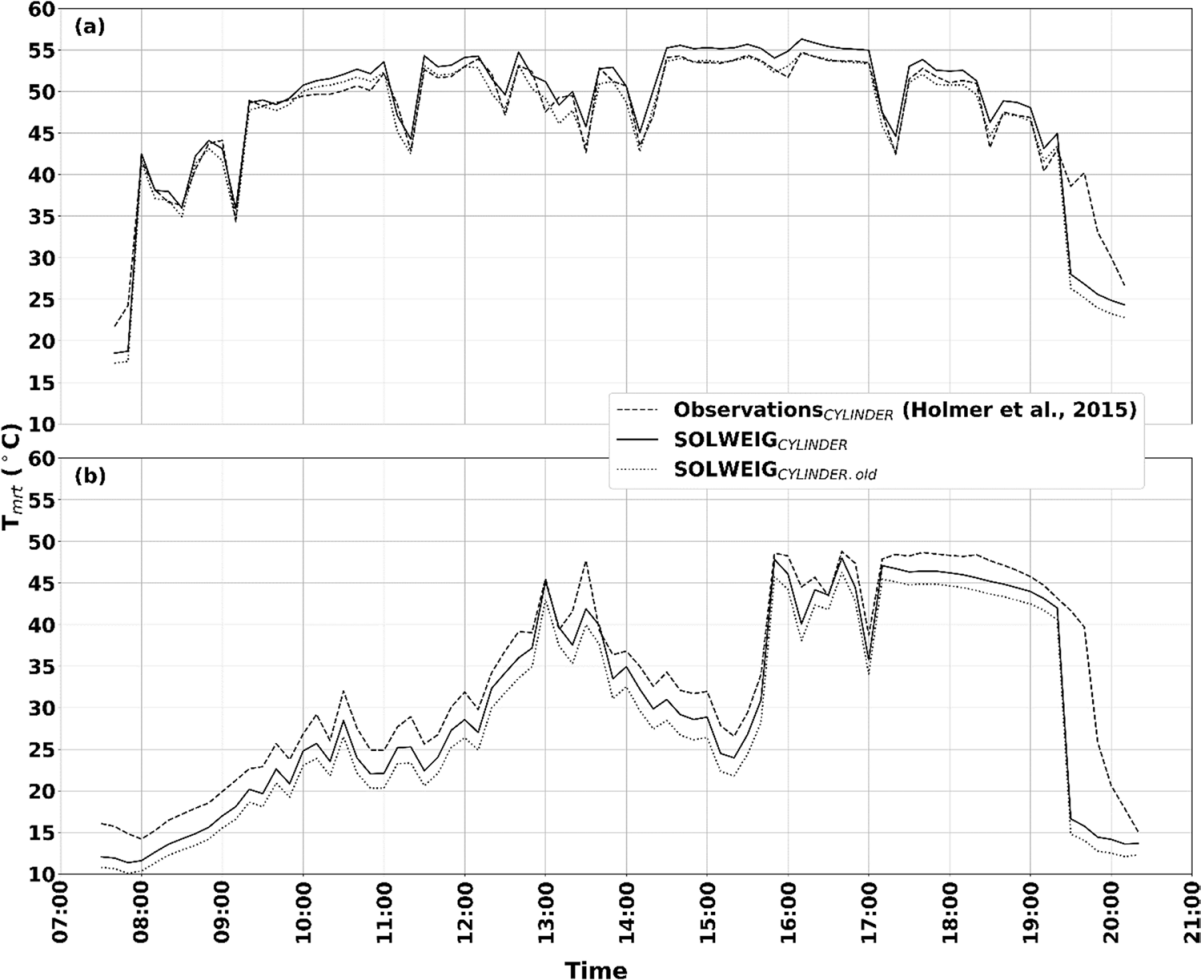
Observed and simulated T_mrt_ for a human represented by a cylinder on **a** a relatively clear day (2021–06-17) and **b** an overcast–semi-cloudy–clear day (2021–06-08). The observed T_mrt_, depicted with dashed lines, is estimated according to Holmer et al. (2015). Simulated T_mrt_ depicted with a solid line is estimated with the new parameterization scheme for longwave radiation, and simulated T_mrt_ depicted with a dotted line is estimated with the old parameterization scheme

Figure 6 shows T_mrt_ for a human represented by a cylinder, where observations are estimated according to Holmer et al. (2015) and simulated T_mrt_ is estimated using the new parameterization scheme for longwave radiation presented in this paper. It should be noted that the sky diffuse shortwave radiation in the method by Holmer et al. (2015) is considered isotropic, whereas in the simulations presented here, sky diffuse shortwave radiation is anisotropic.

T_mrt_ for a relatively clear day is given in Fig. 6a. The simulated T_mrt_ shows slightly higher values compared to the observations. Ignoring T_mrt_ in early morning and late evening when differences depend on shadow patterns (shaded in simulation but sunlit in observations), the largest difference is 3.5 °C at 13:00 (simulation larger than observation). Overestimations can to some extent be explained by the overestimated longwave radiation seen in Fig. 4. On the other hand, diffuse sky shortwave radiation is omitted in observations. Wallenberg et al. (2020) showed that diffuse shortwave radiation increases the radiant load on the vertical of a human (cylinder), suggesting that the observed T_mrt_ shown here could be underestimated. The largest underestimation in the simulations is approximately 1–1.5 °C just after 11:00 and 13:00. The old version of SOLWEIG gives a very good match with observed T_mrt_ throughout the day.

Observed and simulated T_mrt_ for an overcast–semi-cloudy–clear day is presented in Fig. 6b. Here, simulated T_mrt_ is underestimated throughout the day as an effect of the underestimated longwave radiation presented in Fig. 5. Again, as in the previous example, the largest underestimations are visible in the early morning and late evening. Otherwise, the largest underestimations of almost 6 °C and 4.5 C occur at 13:30 and 16:10, respectively, when the sky was semi-cloudy. Except for these examples, a systematic underestimation of around 2–3 °C is evident. Here, simulated T_mrt_ with the old parameterization scheme shows larger underestimations compared with the new parameterization scheme.

**Fig. 7:**
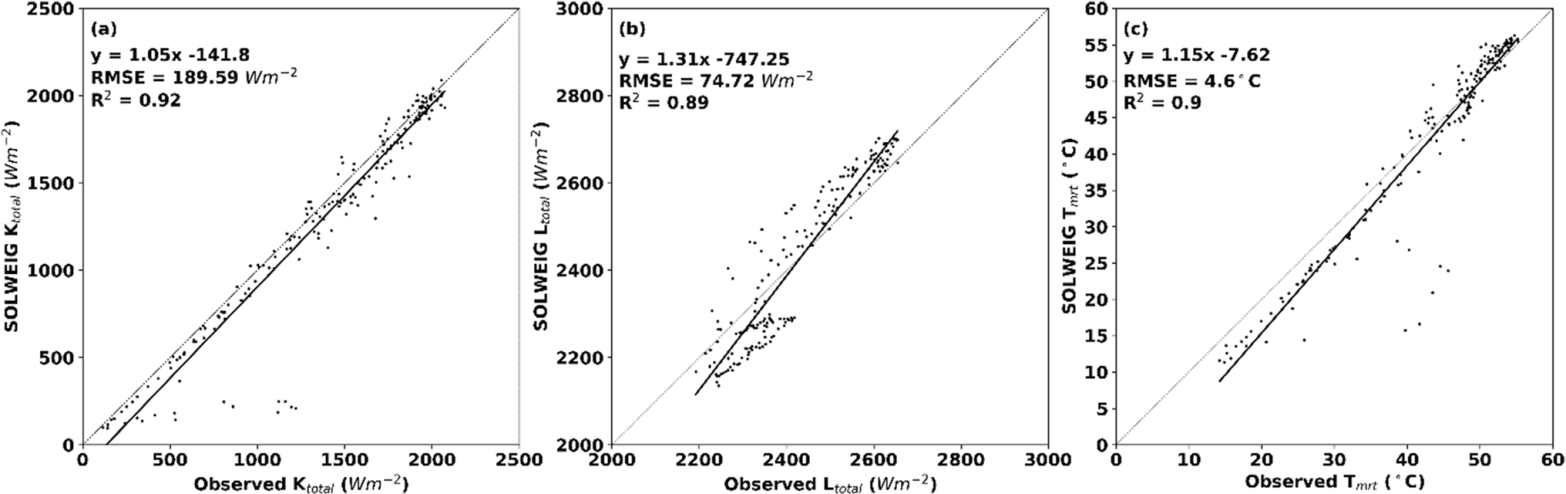
Scatter plots of observed and simulated **a** total shortwave radiation (K_total_, sum of all shortwave radiation fluxes), **b** total longwave radiation (L_total_, sum of all longwave radiation fluxes), and **c** T_mrt_. Data is from 7 days (2018–05-15, 2018–06-08, 2019–07-11, 2020–06-24, 2021–06-08, and 2021–06-17) for the central parts of a square (in and around the white star in Fig. 1)

Scatter plots of observed and simulated total shortwave radiation (K_total_, sum of all six shortwave radiation fluxes), total longwave radiation (L_total_), and T_mrt_ for a human represented by a cylinder using the new parameterization scheme are presented in Fig. 7. Here, all 6 days of field measurements have been used. In Fig. 7a, it is evident that simulated K_total_ correlates well with observed values, indicated by the *R*^2^ values (0.92), even though RMSE is 189.5 Wm^−2^. The relatively high RMSE is explained by the temporal resolution, where on a 60 min temporal resolution, the area can be in shade, in SOLWEIG, even though it was sunlit in observations within the same time span. The sum of longwave radiation (L_total_), given in Fig. 7b, also shows relatively high correlations (*R*^2^ = 0.89 and RMSE = 74.7 Wm^−2^). Underestimations are, however, evident in SOLWEIG when longwave radiation is relatively low and overestimations when longwave radiation is relatively high. This, again, is a product of the surface temperature parameterization, indicating that longwave radiation is overestimated from sunlit surfaces and slightly underestimated from shaded surfaces. In Fig. 7c, the resulting T_mrt_ values are presented. Here, patterns are similar to those seen in the scatter plot for longwave radiation, with underestimations at low T_mrt_ and a small overestimation at high T_mrt_. Nevertheless, the correlation is high (0.9), and RMSE is relatively low (4.6 °C).

### Division of sky into patches—comparison of anisotropic and isotropic skies

**Fig. 8:**
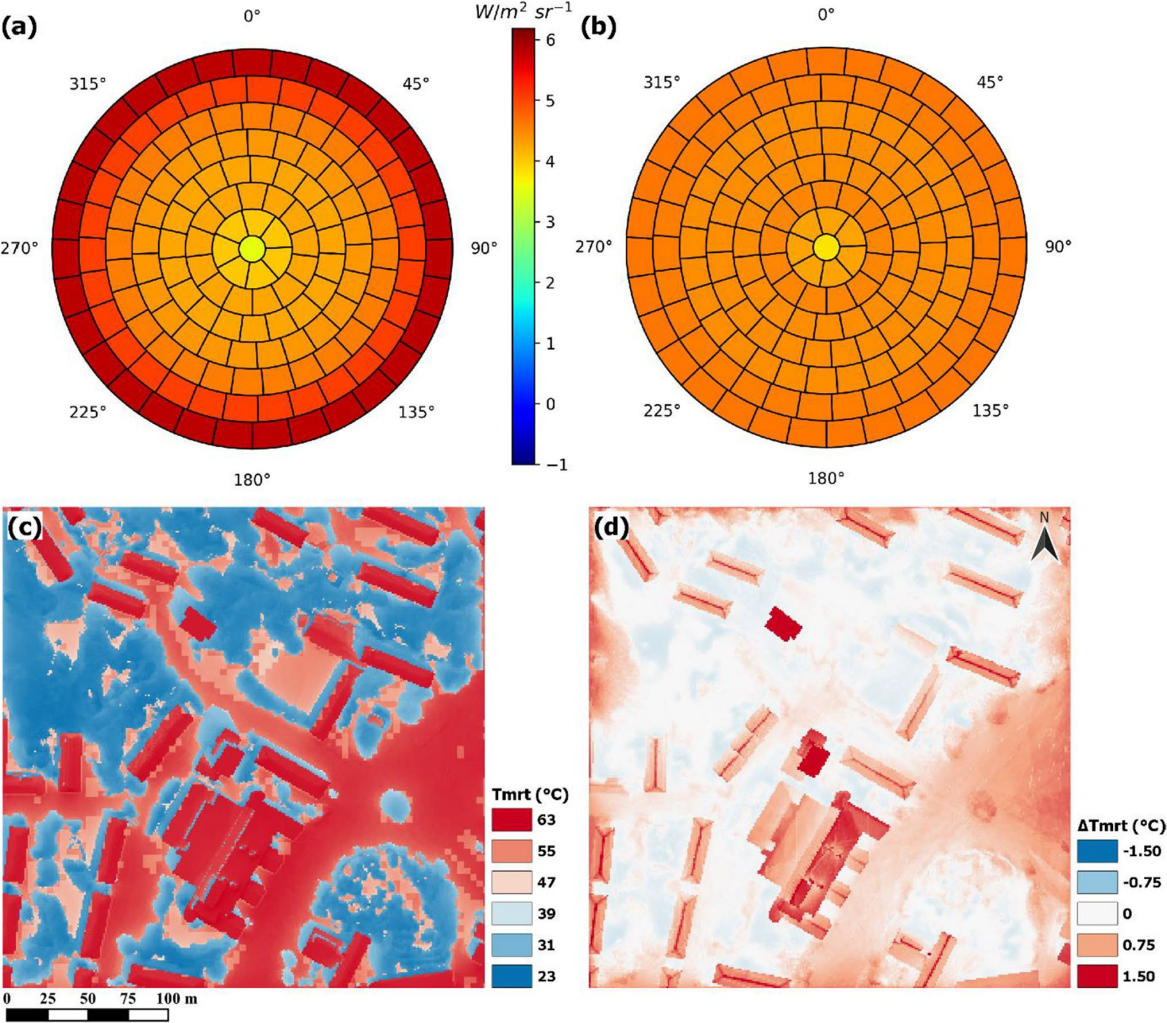
Figure showing output from SOLWEIG for 2021–06-17 12:10 LST, where **a** shows longwave irradiance from an anisotropic sky and **b** from an isotropic sky, normal to a surface. Figure **c** shows T_mrt_ for a human represented by a cylinder with an anisotropic sky for longwave radiation and **d** the difference in T_mrt_ (cylinder) for the corresponding time with an isotropic sky (anisotropic sky–isotropic sky)

Sky longwave radiation distributed in the patches for two simulated skies on 2021–06-17 12:10 LST is presented in Fig. 8. In Fig. 8a, the sky is anisotropic, and sky longwave radiation is increasing with zenith angle, reaching its maximum at the outer band, closest to ground surface. Figure 8b shows longwave radiation for a sky where emissivity distributed into each patch is equal, resulting in an isotropic sky only depending on the solid angles of each patch. Here, the center patch has a slightly smaller solid angle, producing a lower amount of emitted longwave radiation.

An output raster of T_mrt_ simulated with the anisotropic sky (seen in Fig. 8a) for a human represented by a cylinder is presented in Fig. 8c. Here, common patterns are visible, with high T_mrt_ in open areas and in front of sunlit south facing facades. Low T_mrt_ is evident in shade from trees and buildings. A comparison of the output in Fig. 8c and an output with a sky vault divided into patches but with isotropic sky longwave radiation (Fig. 8b) is presented in Fig. 8d. Thus, the only difference in model settings between the two simulations are the skies seen in Fig. 8a, b. The results given in Fig. 8d reveal that some open areas, e.g., the square where observations were carried out, have less sky longwave radiation with an anisotropic sky, whereas areas underneath, e.g., trees, have more. These differences are marginal. Nevertheless, this is explained by exposure to a colder sky (lower emissivity) from lower zenith angles with the anisotropic sky compared to the isotropic sky (see Fig. 8a, b). Underneath the trees, on the other hand, exposure is increased as the anisotropic sky has higher amounts of longwave radiation emitted from high zenith angles. This becomes obvious in open areas (high SVF), where exposure to the warm sky (high emissivity) at high zenith angles results in a higher estimated T_mrt_ with the anisotropic sky, up to 1.5 °C.

Two more comparisons were made although not included in the main body of the text. The first one was for T_mrt_ between the SOLWEIG simulation seen in Fig. 8c and a SOLWEIG simulation with the old parameterization scheme based on SVF (Lindberg et al. 2008, 2016) and is available as Online Resource 3. Here, higher T_mrt_ of up to 2–3 °C is evident especially in front of sunlit walls as an effect of increased exposure to sunlit facades. Lower estimations, on the other hand, are also quite substantial and can be as large as − 3 °C underneath trees (high sky obstruction, SVF < 0.3).

The second comparison was conducted between a SOLWEIG simulation with patches utilizing isotropic sky and a SOLWEIG simulation adopting the old parameterization scheme based on SVF (not included). This comparison had similar results as in Online Resource 3, indicating that the differences between the new and old parameterization schemes are attributable mainly to the implementation of the patches.

## Discussion

We have presented a new parameterization scheme for longwave radiation originating from the upper hemisphere as seen from a human represented by a cylinder or standing box. The new scheme has made it possible to differentiate between longwave radiation emanating from the sky, vegetation, and sunlit or shaded building surfaces. Furthermore, a model for anisotropic sky longwave irradiance is included.

The results given in Figs. 4 and 5 show a good correlation between simulated and observed longwave radiation for both days. The simulated down-welling longwave radiation (L_down.new_) fits well with observed values (L_down.obs_), except for the afternoon. This implies that the model for global sky emissivity by Prata (1996) and the model for anisotropic sky emissivity by Martin and Berdahl (1984a) is a fitting combination during both clear (2021–06-17) and cloudy (2021–07-08) conditions, even though ${\varepsilon }_{\mathrm{sky}}$ is slightly underestimated during the cloudy day. Furthermore, the results from 2021–07-08 show that the correction of sky emissivity during cloudy conditions using CI and cloud fraction (Crawford and Duchon 1999) is satisfying. There are, however, under and overestimations in the remaining longwave radiation fluxes. On the clear day, for example, there are noticeable overestimations in the lateral longwave radiation. These overestimations are reflected in T_mrt_. Except for the possible factors that could explain parts of this (offset in patches due to a possibly inaccurate DSM and sunlit walls down to ground level), this can be explained by the parameterization of surface temperatures (both sunlit building facets and ground surface). Part of the lateral longwave radiation consists of radiation emitted from the ground surface. The parameterization scheme for sunlit surface temperatures in SOLWEIG is based on the works by Bogren et al. (2000). Here, emissivity and surface temperature are determined for each land cover type (e.g., cobble stone, asphalt, and grass), established from measurements of difference in surface temperature (T_s_) and air temperature (T_a_) (T_s-a_) by Bogren et al. (2000) and Lindberg et al. (2016). T_s_ is expected to peak shortly after maximum solar elevation. The initial T_s_ (valid for shaded surfaces) is estimated from T_a_. In sunlit conditions T_s-a_ is added to the initial T_s_, creating a sunlit surface temperature. T_s-a_ follows a sinusoidal curve based on a given time when it will peak in relation to sunrise (see Eqs. 11 and 12 in Lindberg et al. 2008), e.g., 13:00 as is the case for the location of observations in this study. In addition, T_s_ is depending on CI (between 0 and 1, where 1 indicates clear conditions and 0 complete overcast, i.e., darkness (see Crawford and Duchon (1999) for details). Simply, T_s-a_ is multiplied by CI. Simulated T_mrt_ and longwave radiation show a good correlation with observations on the clear day up until around this time, after which it is overestimated. This indicates that the parameterization scheme has difficulties simulating T_s_ subsequent to peak T_s-a_. For 2021–06-08, on the other hand, T_mrt_ is underestimated, especially during overcast or partly cloudy conditions. Since T_s_, in addition, is governed by CI, which is low during overcast or cloudy conditions, ground and building surfaces are exposed to low amounts of shortwave radiation even though the incoming diffuse shortwave radiation is 300–400 Wm^−2^. When weather conditions become clear and CI increases, the correlation of longwave fluxes and T_mrt_ increases, and underestimations are reduced. Similar conclusions can be drawn from the scatter plots, where the slope for total shortwave radiation is close to 1, whereas total longwave radiation has a larger slope (1.31), underestimating at low radiation fluxes (shade) and overestimating at high fluxes (sunlit). The advantage with the current parameterization of sunlit T_s_ is that it is very fast and suitable for a user-friendly 2D modeling approach. The drawback, here, is that it overestimates in the afternoon. The peak surface temperature likely also differs depending on the direction of the wall. Under anticyclonic weather conditions, an east facing façade would, for example, peak in the morning, whereas a west facing façade would peak in the afternoon. In the current parameterization scheme, this is omitted and all wall surfaces peak at the same time, regardless of direction. Thus, the combined effect of a potential misestimate in building patches to the east (where buildings are located), the fact that building wall surfaces can be sunlit down to ground level and that the parameterization scheme for surface temperatures is overestimating in the afternoon results in an overestimated T_mrt_. The potential misestimate in buildings patches is if anything minor. This emphasizes the hypothesis that the parameterization scheme for T_s_ is the main explanation for the overestimations. The largest offsets in longwave irradiance are noticeable from directions where buildings are located (east, north, and south) and for the most part when these building surfaces are sunlit, from midday forward that is. East stands out with the largest offset. This is also the direction where most buildings are located. Longwave irradiance from the west where there are no influencing building facades shows an overestimation of ~ 10 Wm^−2^, although stable throughout the day. This is another indication that the T_s_ for sunlit building facades is the source of overestimations. A new and promising parameterization scheme for T_s_ is under development and will be included in a future version of SOLWEIG. Another potentially influencing factor is that all building surfaces are considered the same, while in fact, they consist of different materials (e.g., concrete, stone, and glass to mention a few) with different emissivity’s and other thermal properties. These differences are overlooked, or calculations would otherwise be too complex, and SOLWEIG would lose one of its advantages that is its speed.

Gal and Kantor (2020) recommended an introduction of a scheme for the directionality of sunlit and shaded surfaces into SOLWEIG, which we here, successfully, demonstrate. This new scheme has made it possible to calculate the radiant load on a human represented by a cylinder, as we now know the amount of longwave radiation originating from each of the 153 different directions. Continuing, the inclusion of an anisotropic sky for longwave irradiance shows that differences to that of an isotropic sky can be as high as 1.5 °C T_mrt_ in open areas under the meteorological conditions presented here. Nahon et al. (2019) calculated T_mrt_ for a cold night in Montreal, Canada, and estimated a 3.5 °C higher T_mrt_ with an anisotropic sky compared to an isotropic. Their results are in line with our results, showing that an anisotropic sky increases the exposure on the vertical facet of a human represented by a box or cylinder to the relatively warmer sky closer to the ground compared to an isotropic sky. However, the largest differences with the new parameterization scheme come from the implementation of the patches. Comparisons with the old parameterization scheme in SOLWEIG (see Lindberg et al. (2008; 2016) for old parameterization scheme) reveal that T_mrt_ in areas with high sky obstruction, e.g., under trees, is reduced by as much as 3 °C. Gal and Kantor (2020) showed overestimations under trees of up to 7 °C, and Lindberg and Grimmond (2011) showed overestimations of ~ 5 °C, indicating that our reduction presented here is an improvement of the SOLWEIG model. In the previous version of SOLWEIG, all areas with SVF > 0 have sunlit surfaces. With the new parameterization scheme, sunlit building surfaces can be obstructed by, e.g., a tree canopy. Furthermore, areas in front of sunlit facades show increases in T_mrt_ of up to 2–3 °C from increased exposure to warm sunlit surfaces. The division of the hemisphere into 153 patches has made it possible to differentiate between sky, vegetation, and sunlit/shaded building surfaces. This has enabled the determination of the direction of longwave irradiance from the corresponding surfaces. Thus, it is now easier to determine and improve errors such as over or underestimations from, e.g., sunlit and shaded building surfaces seen in this paper.

In the study by Rykaczewski et al. (2021), differentiation between direct and diffuse solar radiation (Holmer et al. 2015) using 3D integral measurements (Höppe 1992) led to more realistic results in radiant load on parts of a detailed manikin (as opposed to a cylinder) obstructed from the direct solar beam through self-shadowing. Since their results were in an unobstructed setting, the authors concluded that the model should be evaluated in complex urban settings, including anisotropic sky diffuse shortwave irradiance and anisotropic longwave radiation for a more realistic depiction of radiation patterns. In this paper, we estimate T_mrt_ with knowledge of the position of the sun as well as anisotropic sky diffuse shortwave radiation and anisotropic sky longwave radiation emanating from 153 different parts of the sky vault. Furthermore, longwave radiation originating from vegetation and buildings can be estimated for 153 directions if the sky is obstructed by any of the respective surfaces. Future improvements include implementing the patches for reflections of shortwave radiation on vegetation and buildings and the upward fluxes of shortwave and longwave radiation from the ground. Thus, the SOLWEIG model is closing in on including directionality for all shortwave and longwave radiation fluxes.

Future work includes further development of the surface temperature parameterization scheme to decrease the general overestimation seen in the afternoon. Furthermore, field measurements should be conducted at locations where large differences between the new and old versions of SOLWEIG were seen, e.g., under trees and close to walls.

## Conclusion

We have presented a new parameterization scheme where the upper hemisphere has been divided into 153 patches, incorporating lateral and down-welling longwave radiation fluxes from sky, vegetation, as well as shaded and sunlit building surfaces. The following conclusions can be drawn from the model evaluation:

- Simulated longwave radiation (*R*^2^ = 0.89 and RMSE = 74.7 Wm^−2^) and T_mrt_ (*R*^2^ = 0.9 and RMSE = 4.6 °C) correlate well with observed values, suggesting that the model performance is high.
- A more realistic anisotropic sky for longwave radiation, compared to a uniform sky, increases the exposure on the vertical for a human represented by a cylinder. The resulting implication is an increase in radiant load of up to 1.5 °C in T_mrt_, in areas where most of the sky vault is visible.
- Comparison of the patches with the old parameterization scheme reveals decreases in previous overestimations under tree canopies of up to around 3 °C in T_mrt._ Furthermore, radiant load close to sunlit walls shows an increase of up to 2–3 °C.
- The division of the upper hemisphere into 153 patches makes it possible to determine the characteristics surrounding a human in 153 different directions. It is now possible to estimate which of the 153 different directions are sky, vegetation, shaded building surfaces and sunlit building surfaces, with corresponding longwave irradiance.

## Supplementary Information

Below is the link to the electronic supplementary material.

Supplementary file1 (PDF 651 KB)

## Data Availability

The datasets generated during and/or analysed during the current study are available from the corresponding author on reasonable request.
