# Supplementary material for: An anisotropic parameterization scheme for longwave irradiance and its impact on radiant load in urban outdoor settings
Source: Int J Biometeorol. 2023 Feb 24;67(4):633–47. doi: 10.1007/s00484-023-02441-3 (PMC10070231; doi:10.1007/s00484-023-02441-3)
Supplement: Supplementary file 1 — Supplementary file1 (PDF 651 KB) [file 484_2023_2441_MOESM1_ESM.pdf]

Appendix

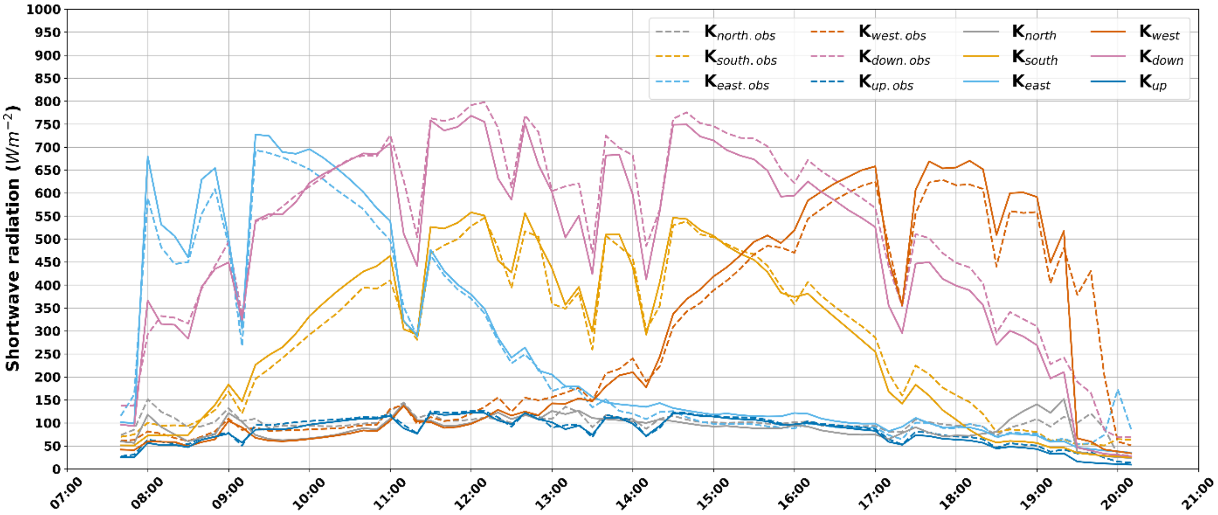

Online resource 1 Shortwave radiation fluxes on 2021-06-17. Dashed lines depicts observations and solid lines simulations.

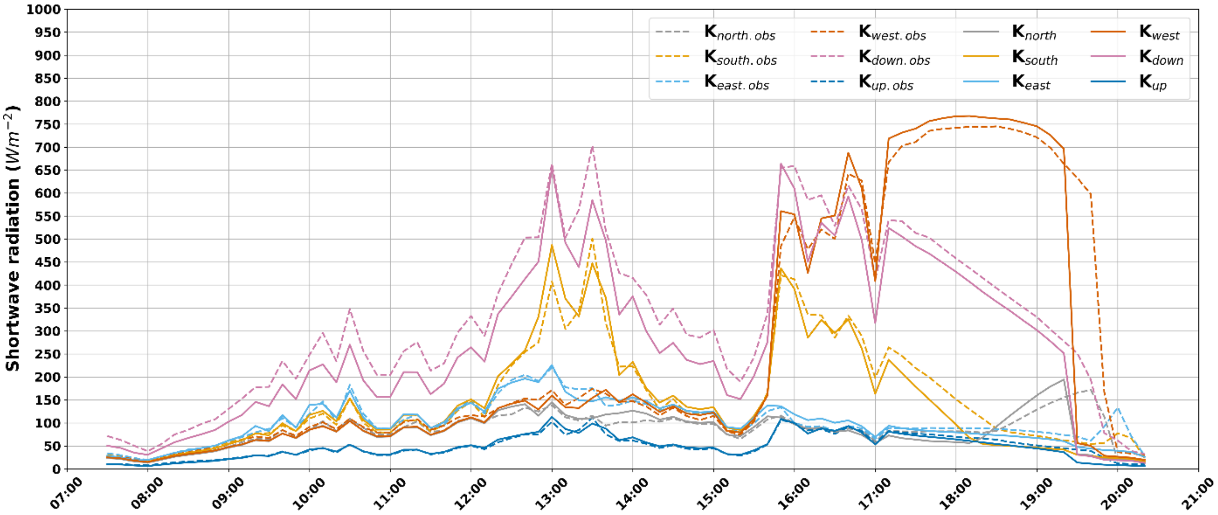

Online resource 2 Shortwave radiation fluxes on 2021-06-08. Dashed lines depicts observations and solid lines simulations.

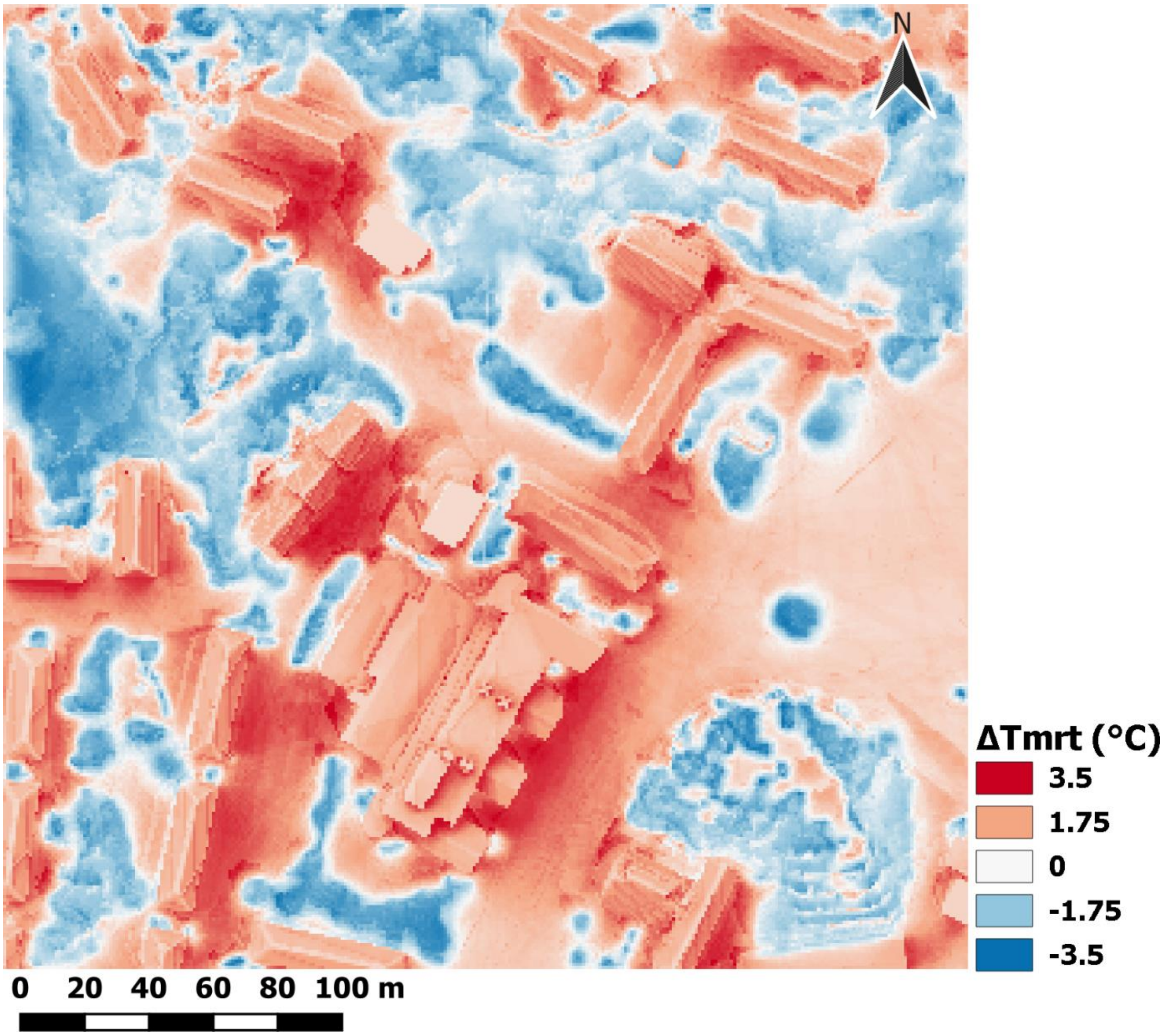

Online resource 3 Map showing difference in mean radiant temperature (T_{mrt}) between new and old versions of SOLWEIG. In the new version of SOLWEIG longwave radiation originating from the upper hemisphere has been divided into 153 patches compared to the old version of SOLWEIG where longwave radiation is estimated from sky view factors. Positive values indicate larger T_{mrt} with the new version of SOLWEIG and negative values demonstrate areas where T_{mrt} is lower.
